# Parametric instability of optical non-Hermitian systems near the exceptional point

**DOI:** 10.1038/srep29709

**Published:** 2016-07-13

**Authors:** A. A. Zyablovsky, E. S. Andrianov, A. A. Pukhov

**Affiliations:** 1 All-Russia Research Institute of Automatics, 22 Sushchevskaya, Moscow 127055, Russia; 2 Moscow Institute of Physics and Technology, 9 Institutskiy per, Dolgoprudny 141700, Russia; 3 Institute for Theoretical and Applied Electromagnetics, 13 Izhorskaya, Moscow 125412, Russia

## Abstract

In contrast to Hermitian systems, the modes of non-Hermitian systems are generally nonorthogonal. As a result, the power of the system signal depends not only on the mode amplitudes but also on the phase shift between them. In this work, we show that it is possible to increase the mode amplitudes without increasing the power of the signal. Moreover, we demonstrate that when the system is at the exceptional point, any infinitesimally small change in the system parameters increases the mode amplitudes. As a result, the system becomes unstable with respect to such perturbation. We show such instability by using the example of two coupled waveguides in which loss prevails over gain and all modes are decaying. This phenomenon enables compensation for losses in dissipative systems and opens a wide range of applications in optics, plasmonics, and optoelectronics, in which loss is an inevitable problem and plays a crucial role.

Recent developments in nanoscience have led to dramatic decreases in system size. The inherent property of such small-size systems is the impossibility of separation of the environment from the system under consideration. For this reason, the investigation of open and, in particular, non-Hermitian systems has been among the main topics of physics over the last decade^1^,^2^,^3^,^4^,^5^,^6^,^7^,^8^.

Non-Hermitian systems are of interest due to their specific properties that are not present in Hermitian systems. In general, the eigenstates of non-Hermitian systems are not orthogonal^4^,^5^,^6^,^7^, in contrast to Hermitian systems. The maximal degree of non-orthogonality is reached at the exceptional point (EP). The EP is a point in the parameter space at which two or more eigenstates coalesce to one eigenstate. As a result, the eigenstates become linearly dependent^4^,^7^ and do not form the basis^9^. The existence of the EP in various types of physical systems such as microwave cavities^10^, optical microcavities^11^,^12^ and chaotic optical microcavity^13^ has been demonstrated. The existence of the EP leads to non-trivial topology of the system spectrum^10^,^14^. In ref. 14 it was shown theoretically that the adiabatic encircling of the EP in the complex plane of system parameters leads to a phase shift of the system state. This phase shift is analogous to the geometrical (Berry) phase in quantum mechanics^9^,^10^. In ref. 10 such a geometric phase was measured for optical microcavity modes when an EP was encircled. The geometrical phase has also been observed in waveguides with spatial changes in parameters^15^. The physical properties of the EP may be useful for sensor applications^11^,^12^.

In recent years, much attention has been paid to the investigation of EPs in PT-symmetric^1^,^2^,^3^,^4^,^5^,^6^,^7^ and quasi-PT-symmetric systems^16^. A system is called PT-symmetric if its Hamiltonian is invariant under the combined operations of parity (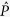) and time (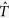) reversal^1^,^2^,^3^. The important property of PT-symmetric systems is that their spectrum may be both real and complex^1^,^2^,^3^. The values of the system parameters at which the transition from real to complex spectrum occurs form the point of phase transition^1^,^2^,^3^,^6^,^7^,^8^. In PT-symmetric systems, the EP and the point of phase transition from the real spectrum to the complex spectrum coincide^7^. Recently, quantum and optical PT-symmetric systems have been actively investigated both experimentaly^16^,^17^,^18^,^19^,^20^,^21^,^22^,^23^,^24^ and theoretically^1^,^2^,^3^,^6^,^7^,^8^,^25^,^26^,^27^,^28^,^29^,^30^,^31^.

Periodic perturbations may lead to instability of the PT-symmetric system when such perturbations shift the system parameters. In this case, the system spectrum becomes complex and some eigenmodes undergo exponential growth^32^,^33^,^34^.

In this work, we investigate an optical non-Hermitian system in which the parameters change periodically, losses exceed gain, and all eigenmodes are decaying. We show that changes to the parameters of this non-Hermitian system may result in increased of eigenmode amplitudes without simultaneously increasing the power of the system signal during the change. The increased amplitudes of the eigenmodes lead to increased amplitudes of the power oscillations. If such perturbations are repeated with the half period of power oscillations, then the amplitudes of the eigenmodes and the power of system signal grow infinitely.

In general, not all parameters perturbations result in increasing of amplitudes of the eigenmodes. However, we demonstrate that when the system is at the EP any infinitesimally small change in the system parameters leads to increasing amplitudes of the eigenmodes. As a result, the system becomes unstable with respect to such perturbations. Thus, a new effect occurs in optical non-Hermitian systems: parametric instability near the exceptional point (PIEP). We illustrate the phenomenon of PIEP in the case of two coupling waveguides with gain and loss when the loss in one waveguide is more than the gain in the other. In this system, the total power may increase infinitely, even when all waveguide modes are always decaying. This phenomenon opens a wide range of applications in optics, plasmonics, and optoelectronics, in which loss is an inevitable problem and plays a crucial role.

## Results

### Non-orthogonality of eigenmodes of non-Hermitian systems: power oscillations and non-exponential transient behavior

Before introducing the concept of PIEP, we review the important properties of non-Hermitian systems. The non-orthogonality of eigenmodes in a non-Hermitian system leads to power oscillation^5^,^6^. This phenomenon is the consequence of the dependence of the power of the system signal on both the amplitudes and (in contrast to a Hermitian system) the phases of the eigenmodes. Hereinafter, we restrict our consideration to optical systems. The following consideration is applicable to the optical modes of microcavities and to the modes of waveguide systems.

For simplicity, we consider the waveguide system with two modes. The electric field envelope in the system in point **r** has the form^6^,^8^,^16^,^17^


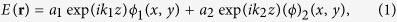


where *a*_*i*_ and *k*_*i*_ are the initial amplitudes and wavenumbers of the modes, respectively; *ϕ*_*i*_(*x*, *y*) is the transverse field distribution in the *i*th mode. The eigenmode amplitudes should be chosen such that 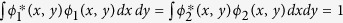. In waveguide systems, the power of the signal is defined as^6^,^35^ 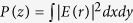.

**Figure 1:**
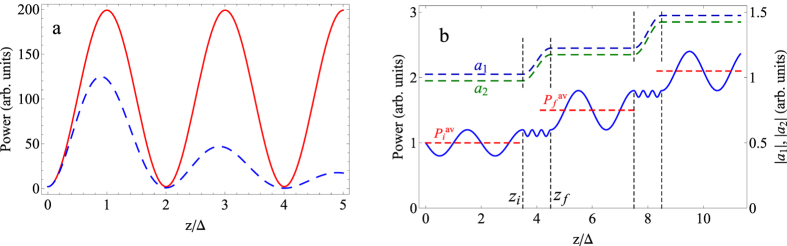
Power oscillations with and without perturbations. (**a**) The dependence of the power in the system on coordinate without perturbation, when system wavenumbers are real (red solid line) and complex (blue dashed line). |*a*_1_|^2^ = 50, |*a*_2_|^2^ = 50, |*A*| = 98.2, *θ* = 0.995*π*, and Im(*k*_1_ + *k*_2_)/*k* = 0.078. (**b**) The dependence of the power (blue solid curve) and amplitudes of eigenmodes (blue and green dashed curves) in the system on coordinate with perturbation. Black dashed vertical lines denote the start and end coordinates of the perturbations. Red horizontal dashed lines denote the average value of the power (diagonal part) between perturbations. Δ = *π*/|Re(*k*_1_ − *k*_2_)| is the half period of oscillations. *a*_1_(*z* = 0) = 2.05, *a*_2_(*z* = 0) = 1.95, *A*(*z* = 0) = 1.6, *θ*(*z* = 0) = 0.

In a non-Hermitian system the eigenmodes are not orthogonal, 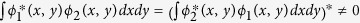. If the wavenumbers of the modes are real, that is, Im*k*_1_ = Im*k*_2_ = 0 (as in a PT-symmetric system below the exceptional point), then the power of the signal has the form


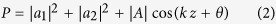


where 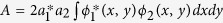, *k* = Re(*k*_1_ − *k*_2_), 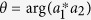 and we suppose that the origin of coordinate is 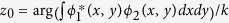, see also Methods. In other words, the power oscillates during propagation; see Fig. 1a and also 6. We designate the diagonal term in (2) |*a*_1_|^2^ + |*a*_2_|^2^ as *P*^*av*^ and the non-diagonal one |*A*| cos(*kz* + *θ*) as *P*^*osc*^. Note that *P*^*av*^ equals the average value of the power during propagation.

If all the wavenumbers are decaying, that is, Im*k*_1_ < 0 and Im*k*_2_ < 0, then the system exhibits non-exponential transient behavior^36^:


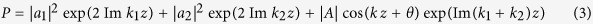


Non-orthogonality of the eigenmodes leads to power oscillations when the total power in the system oscillates during propagation^5^,^6^. Additionally, due to the non-orthogonality of eigenmodes, non-Hermitian systems may exhibit non-exponential behavior^36^ when all the eigenmodes are decaying. In this case, during the first stage, the power increases compared with the initial value and decreases exponentially in the second stage^36^ (Fig. 1a). The increasing power in the first stage is the consequence of the field concentration in the gain layers. The field redistribution in the second stage leads to the exponential decay of the field amplitude^36^.

Note that when gain is absent in the system, the power is also described by Eq. (3). However, it cannot be amplified, which means the system parameters should obey certain conditions^35^.

Non-exponential behavior and power oscillation relate to the non-orthogonality of eigenmodes. The maximal degree of non-orthogonality is reached at the EP. At this point, the spectrum of the system is degenerate, that is, *k*_1_ = *k*_2_ = *k*, and the eigenmodes are equal to each other, i.e., they do not form the basis^37^.

### Parametric amplification in a system with non-orthogonal eigenmodes

Power oscillation, which we describe above, can be used for the parametric amplification of the power. To demonstrate, we expose the system to periodic perturbation, which redistributes the power between diagonal *P*^*av*^ and non-diagonal *P*^*osc*^ terms.

Let the perturbation begin at coordinate *z* = *z*_*i*_ and stop at coordinate *z* = *z*_*f*_ so that the parameters of the system take unperturbed values after perturbation. In addition, we require that this perturbation does not change the power of the system signal. Then in the case in which Im*k*_1_ = Im*k*_2_ = 0, from Eq. (2), we have the following relation:


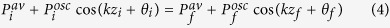


where the subscripts *i* and *f* correspond to the initial and final states. Here, (*kz*_*f*_ + *θ*_*f*_) denotes the phase mismatch between the amplitudes of the first and second eigenmodes after perturbation. The diagonal part of the power after perturbation 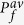 equals


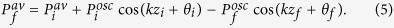


Now, we choose the perturbation such that at initial coordinate *z* = *z*_*i*_ the non-diagonal part is positive, that is, cos(*kz*_*i*_ + *θ*_*i*_) > 0, while at *z* = *z*_*f*_, the non-diagonal part is negative, that is, cos(*kz*_*f*_ + *θ*_*f*_) < 0. In this case, the diagonal part of the power at the end of the perturbation will be larger than at the beginning of the perturbation. The increase in the diagonal part of the power is accompanied by increases in the amplitude of the system eigenmodes *a*_1_ and *a*_2_. At the same time, the total power of the system signal does not change (see the dynamics in Fig. 1b from *z*_*i*_ to *z*_*f*_).

**Figure 2:**
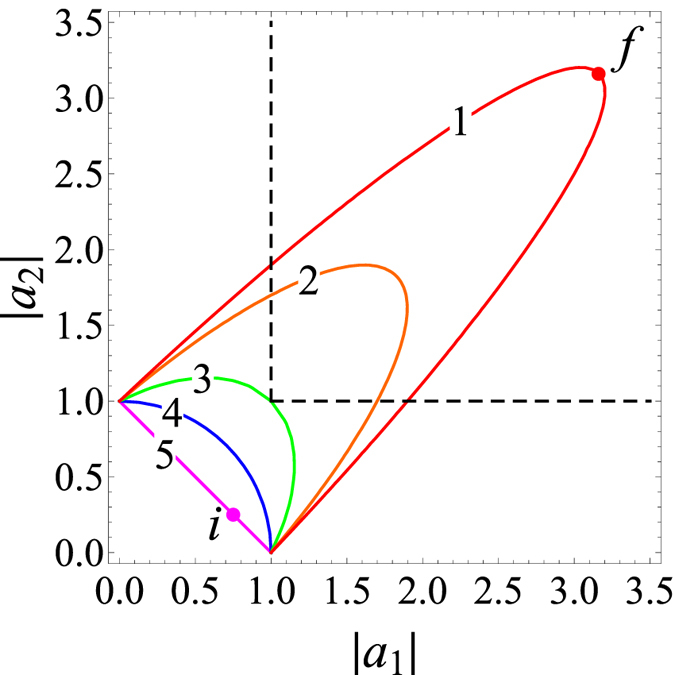
System dynamics in phase space. Possible values of |*a*_1_| and |*a*_2_| at fixed power and phase shift. (1) cos(*kz* + *θ*) = −0.95, (2) *cos*(*kz* + *θ*) = −0.85, (3) *cos*(*kz* + *θ*) = −0.5, (4) *cos*(*kz* + *θ*) = 0, (5) *cos*(*kz* + *θ*) = 1. For all lines, *P* = 1. Point *i* on line 5 corresponds to the initial state of the system before perturbation, and point *f* on line 1 corresponds to the final state of the system after perturbation. In the region bounded by the dashed lines, the amplitudes of both eigenmodes are more than the maximum possible values of the amplitudes of the eigenmodes in the Hermitian system.

We illustrate the influence of the interference term cos(*kz* + *θ*) on the power of the system signal in Fig. 2, where possible values of |*a*_1_| and |*a*_2_| at fixed values of power and phase shift are shown. The most favorable case for increasing the amplitudes of the eigenmodes is when cos(*kz*_*i*_ + *θ*_*i*_) = 1, cos(*kz*_*f*_ + *θ*_*f*_) = −1 and |*a*_1_| ≈ |*a*_2_|. At a fixed value of the total power of the system signal, the amplitudes of the eigenmodes are minimal when cos(*kz* + *θ*) = 1 and maximal when cos(*kz* + *θ*) = −1 and |*a*_1_| = |*a*_2_| (Fig. 2). Thus, the conditions cos(*kz*_*i*_ + *θ*_*i*_) = 1 for the initial state of the system (point i on line 5 in Fig. 2) and cos(*kz*_*f*_ + *θ*_*f*_) ≈ −1, |*a*_1_| ≈ |*a*_2_| for the final state (point f on line 1 in Fig. 2) are optimal for increasing the eigenmode amplitudes.

Now, if we repeat such a perturbation with a period equal to an odd number of half periods of power oscillation, e.g., Δ = *π*/|Re(*k*_1_ − *k*_2_)|, we will have permanent growth of the power in the system (Fig. 1b).

**Figure 3:**
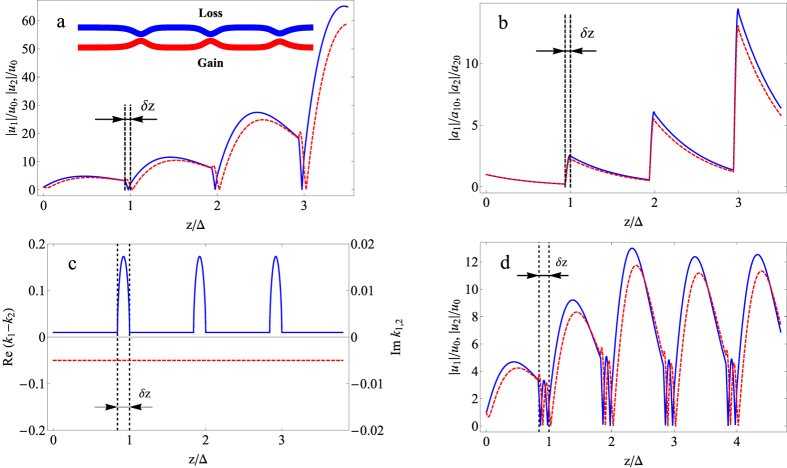
Parametric instability near the exceptional point. (**a**) The dependence of the field amplitude in the first waveguide, |*u*_1_|/*u*_0_ (blue solid line), and in the second, |*u*_2_|/*u*_0_ (red dashed line), on the coordinate *z* along the waveguides. Here, *u*_0_ = *u*_1_(*z* = 0) = *u*_2_(*z* = 0) is the initial amplitude in both waveguides. Inset: system under consideration. (**b**) The dependence of the amplitudes of the first (blue solid line) and second (red dashed line) eigenmodes, |*a*_1_|/*a*_10_ and |*a*_2_|/*a*_20_, on the coordinate *z* along the waveguides. Here, *a*_10_ = *a*_1_(*z* = 0) and *a*_20_ = *a*_2_(*z* = 0) are the initial amplitudes of the first and second eigenmodes. (**c**) The difference between the real parts of the wavenumbers (blue solid line) and the imaginary parts of the wavenumbers (red dashed line). (**d**) The dependence of the field amplitudes |*u*_1_|/*u*_0_ and |*u*_2_|/*u*_0_ in the nonlinear waveguides with *α* = 10^−4^ (see Eq. (12)) on the coordinate *z* along the waveguides. The distance between the waveguides changes with the period Δ = *π*/|Re(*k*_1_ − *k*_2_)|, perturbation length *δz* ≪ Δ, *g*^2^ = 2.5⋅10^−3^, *γ* = 5⋅10^−3^ and |*κ*(*z*)|^2^ = 1.01*g*^2^ outside the perturbation region and 1.01*g*^2^ ≤ |*κ*(*z*)|^2^ ≤ 4*g*^2^ inside the perturbation region.

The power in the system may grow even when all the wavenumbers at any coordinate have negative imaginary parts, that is, Im*k*_1_ < 0, Im*k*_2_ < 0. In this case, perturbation of the system redistributes the power between diagonal and non-diagonal terms, which leads to increased eigenmode amplitudes, as in the previous case. The total power of the system signal increases in the coordinate interval when the parameters do not change and all the wavenumbers are constant and have negative imaginary parts (Im*k*_1_ < 0, Im*k*_2_ < 0), whereas during perturbation, the power decreases. Conversely, eigenmode amplitudes increase during perturbation and decrease otherwise (see also next section and Fig. 3a,b).

### Describing suitable perturbation

As mentioned above, to increase the power of the signal, the following conditions should be satisfied: cos(*kz*_*i*_ + *θ*_*i*_) ≈ 1, cos(*kz*_*f*_ + *θ*_*f*_) ≈ −1, |*a*_1_| = |*a*_2_|. In a optical non-Hermitian system, such perturbation can be simply achieved near the EP, where two eigenmodes almost coincide with each other. Let us introduce the normalized state *ϕ*_⊥_(*x*, *y*), which is orthogonal to the first of the eigenmode: 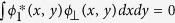, 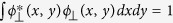. Then, the electric field of second eigenmode *ϕ*_2_(*x*, *y*) can be written in the form 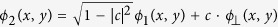, where 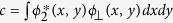. Note that near the EP, we have |*c*| ≪ 1 (in the EP *ϕ*_1_(*x*, *y*) = *ϕ*_2_(*x*, *y*)). At *z* = *z*_*i*_, let the system state be


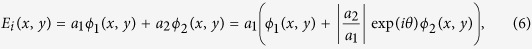


where 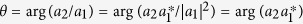: that is, we have the same phase *θ* as in Eq. (2).

Consider an external perturbation that changes the system parameters such that at *z* = *z*_*f*_, the system state takes the form


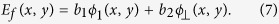


The expansion *E*_*f*_ (*x*, *y*) in the system eigenmodes gives


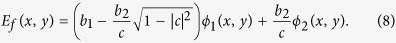


From (8), we see that when the perturbation is such that |*b*_2_/*b*_1_| ≪ *c*, the amplitudes of the eigenmodes after perturbation (expansion coefficients of *E*_*f*_ (*x*, *y*) in (8)) are approximately equal by modulus and have opposite signs:


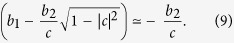


Equations (8) and (9) show that the final state corresponds to the phases cos(*kz*_*f*_ + *θ*_*f*_) ≈ −1, |*a*_1_| ≈ |*a*_2_|. Thus, if the chosen initial state corresponds to the phases cos(*kz*_*i*_ + *θ*_*i*_) ≈ 1, then the perturbation satisfies all conditions for the observation of power amplification. After a half period of power oscillation, Δ = *π*/|Re(*k*_1_ − *k*_2_)|, we have again *cos*(*kz* + *θ*) = 1, and the perturbation may be repeated. Near the EP, *c* ≈ 0 and condition |*b*_2_/*b*_1_| ≫ |*c*| are satisfied automatically. It should be emphasized that this condition means that almost any perturbation increases the eigenmode amplitudes.

Note that in the considered case, the changing of the system parameters is non-adiabatic. The case of adiabatic perturbation near EP has been considered in ref. 38.

The non-Hermitian character of the system is very important. Indeed, in the Hermitian system all eigenmodes are orthogonal (*c* = 1), and the power of the system signal does not depend on the phase mismatch between the amplitudes of the eigenmodes.

It should be noted that such tuning of perturbation to increase the power resembles a situation used in Hermitian systems to obtain parametric resonance. However, this resemblance is superficial. First of all, in our case, increasing power occurs when the parameters do not change, although the eigenmode amplitudes increase when the parameters change (see Fig. 1b, the dynamics from *z*_*i*_ to *z*_*f*_). By contrast, in the case of parametric resonance, the power and eigenmode amplitudes increase simultaneously with the changes in the parameters. Second, in a non-Hermitian system near the EP, almost any perturbation results in increasing eigenmode amplitudes, whereas in the case of parametric resonance in a Hermitian system, it is necessary to fulfill certain conditions^39^. These key points allow us to consider this effect of *parametric instability near the exceptional point* (PIEP) as a new phenomenon and a distinctive feature of optical non-Hermitian systems near the EP.

To summarize, in a optical non-Hermitian system, a certain change in the system parameters leads to increasing eigenmode amplitudes. In EP, eigenmode amplitudes increase upon any change. Such an increase arises due to power transfer from the oscillating non-diagonal part to the diagonal part. As a result, the average power and the amplitude of the power oscillation also increase.

If we change the system parameters periodically with a period equal to an odd number of half periods of power oscillation, e.g., Δ = *π*/|Re(*k*_1_ − *k*_2_)|, then we will have permanent growth of the average power, limited only by nonlinear effects.

### PIEP in an optical system

Above, we define the conditions under which PIEP takes place. Now, we provide an example of a system with PIEP. Let us consider a system consisting of two coupling waveguides in which the real parts of the waveguide refractive index are equal, that is, Re *n*_1_ = Re *n*_2_, while the imaginary parts are different and have opposite signs, that is, Im *n*_1_ > 0, Im *n*_2_ < 0; loss exceeds gain, |Im *n*_1_| ≥ |Im *n*_2_|^16^; and the distance between waveguides changes with the period Δ_var_ (see Fig. 3a).

Let the *z*-axis be directed along the waveguides. In coupled-mode theory^8^,^16^,^40^, the amplitudes of the electric field in the first and second waveguides are written in the form *E*_1,2_(*x*, *y*, *z*) = *F*_1,2_(*x*, *y*)*u*_1,2_(*z*). The dependence of the *u*_1_(*z*) and *u*_2_(*z*) is described by the following system:


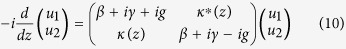


where *β* is the real part of the wavenumbers. For symmetry, we introduce half of the sum of the imaginary parts of the wavenumbers *γ* (which corresponds to common background damping or amplification) and half of the difference between the imaginary parts *g*. Here, *κ*(*z*) is the coupling constant, which depends on the distance between waveguides.

The wavenumbers of the system (10) are 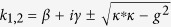, and the dependence of the field on the coordinate *z* in the system where *κ* does not depend on coordinate *z* is as follows:


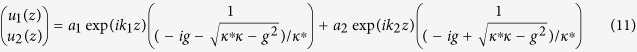


where constants *a*_1_ and *a*_2_ are determined by the initial conditions. The total electric field in the system *E*(*x*, *y*, *z*) = *E*_1_(*x*, *y*, *z*) + *E*_2_(*x*, *y*, *z*) may be presented as in Eq.(1) with 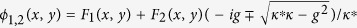.

If |*κ*|^2^ ≥ *g*^2^, then the imaginary parts of all wavenumbers are positive, Im*k*_1,2_ = *γ* > 0, and all eigenmodes are decaying.

When the coupling constant |*κ*|^2^ ≥ *g*^2^ does not depend on coordinate *z*, the power of the signal *P* non-exponentially depends on the distance. In the first stage, the power grows due to non-orthogonal eigenmodes of the system, and then this growth is changed by the exponential decay. As a result, the power of the signal tends to zero when *z* → ∞; see also Fig. 1a and ref. 36.

We consider the case when the distance between waveguides is constant except for the region with length *δz* (a perturbation length), which is repeated with period Δ_var_ ≫ *δz*. In this situation *parametric instability near the exceptional point* may be observed. PIEP leads to the growth of the power of the signal in the system (*P* = |*u*_1_|^2^ + |*u*_2_|^2^) (Fig. 3a). Note that we considered only the case when the condition |*κ*|^2^ ≥ *g*^2^ is satisfied at all *z*, i.e., Im*k*_1,2_ > 0 (Fig. 3c), and all eigenmodes decay. The power growth occurs even in this case (Fig. 3a) due to the parametric instability of the system near the exceptional point. Note that the power of the signal (*P* = |*u*_1_|^2^ + |*u*_2_|^2^) before perturbation *P*_*i*_ is no less than the power after perturbation *P*_*f*_ (see Fig. 3a), although the amplitudes of the eigenmodes after perturbation are larger than before one (Fig. 3b). Thus, within the perturbation region, the total power of the signal decreases while the amplitudes of the eigenmodes increase, and vice versa outside the perturbation region. This behavior validates the difference between PIEP and ordinary parametric resonance in a Hermitian system.

**Figure 4:**
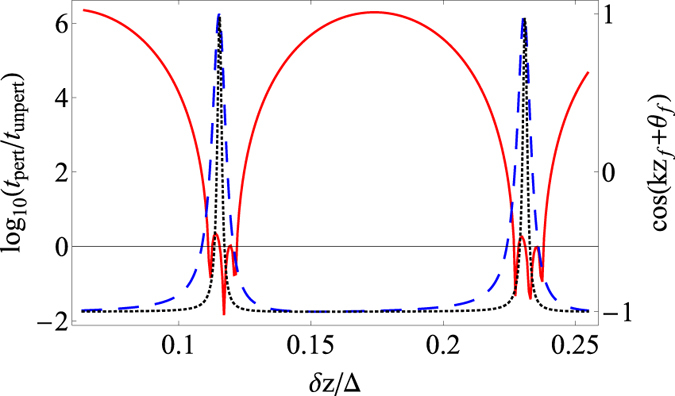
Instability region. The dependence of the ratio (logarithmic scale) of the transmission coefficient *t* = (|*u*_1_(*L*)|^2^ + |*u*_2_(*L*)|^2^)/(|*u*_1_(0)|^2^ + |*u*_2_(0)|^2^) of perturbed *t*_*pert*_ to unperturbed *t*_*unpert*_ systems on the perturbation length *δz* (red solid line) and the dependence of the value cos(*kz*_*f*_ + *θ*_*f*_) after perturbation on the perturbation length *δz* (blue dashed line). For both lines (red and blue) |*κ*|^2^ = 1.01*g*^2^, *u*_1_(0) = *u*_2_(0). Black dotted line: the dependence of the value *cos*(*kz*_*f*_ + *θ*_*f*_) after perturbation on the perturbation length *δz*, in the case of |*κ*|^2^ = 1.001*g*^2^. The EP corresponds to the case when |*κ*|^2^ = *g*^2^. Other parameters are the same as in Fig. 3.

To observe PIEP, before perturbation the state should have the value cos(*kz*_*i*_ + *θ*_*i*_) ≈ 1, whereas after perturbation, the state should have the value *cos*(*kz*_*f*_ + *θ*_*f*_) ≈ −1. Here, *kz* + *θ* is the phase difference between the amplitudes of the eigenmodes. In the previous section, it has been shown that near the EP, any perturbation is suitable. This statement is confirmed by numerical simulation: condition *cos*(*kz*_*f*_ + *θ*_*f*_) ≈ −1 is satisfied for a wide range of perturbation lengths *δz* (Fig. 4). Moreover, when the system parameters tend to the EP, the suitable perturbation length increases (Fig. 4).

For a perturbation length *δz* for which *cos*(*kz*_*f*_ + *θ*_*f*_) ≈ −1, the transmission coefficient of the perturbed system is larger than for the unperturbed system (Fig. 4). The opposite situation is realized only for a small region *δz* in which *cos*(*kz*_*f*_ + *θ*_*f*_) ≈ 1 (see Fig. 4). It is important that the transmission coefficient of the perturbed system is larger even if the period of perturbation Δ_var_ does not coincide with its optimal value Δ = *π*/|Re(*k*_1_ − *k*_2_)|. Figure 4 shows that power amplification occurs at almost any perturbation length. Thus, it is possible for the power to be unlimitedly amplified in a system where loss is dominated by gain and all eigenmodes decay. This effect is a particular case of the *parametric instability near the exceptional point*, whose general properties are described in the previous sections.

It should be noted that system parameters at which PIEP occurs are experimentally achievable. In ref. 16, a quasi-PT-symmetric system consisting of two coupled waveguides has been realized for the cases |*κ*|/*g* < 2 and |*κ*|/*g* ≫ 1. This system is suitable for achieving PIEP.

### Nonlinear effects

All previous consideration was focused on a linear system. However, when the amplitude *u*_1,2_ increases, it is necessary to consider nonlinear effects^32^,^34^,^41^,^42^,^43^,^44^,^45^ that may be connected, for example, to the saturation of the active medium. For this purpose, let us suppose that the amplification and losses in each waveguide depend on the field amplitude in the following equation^46^,^47^


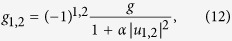


where *α* is the nonlinearity coefficient. Following from the results of the numerical simulation, PIEP also occurs in this case (Fig. 3d). The difference is that the power grows to some finite value that depends on the perturbation parameters *δz* and Δ_var_. We provide a more careful analysis in a forthcoming paper.

## Discussion

In this work, we show that perturbation of the parameters of optical non-Hermitian system may result in increased eigenmodes amplitudes without increasing the power of the system signal during the perturbation. The increasing amplitude of the eigenmodes leads to increasing amplitudes of the power oscillations. We show that the maximal value of the amplitude increase results when the perturbation occurs at the moment when the power of the system signal has its maximum during oscillations. If such perturbations are repeated with the half period of power oscillations, the eigenmode amplitudes and power of the system signal can grow infinitely. This increase may occur even when all system eigenmodes are decaying. In the general case, not all parameter perturbations result in increasing eigenmode amplitudes. However, we demonstrate that when the system is at the exceptional point, any perturbation that changes the parameters of the system leads to increasing eigenmode amplitudes. As a result, the system becomes unstable with respect to such perturbation. This phenomenon is a new effect in optical non-Hermitian systems: parametric instability near the exceptional point (PIEP).

We illustrate the phenomenon of PIEP in the case of two coupling waveguides with gain and loss, when the loss in one waveguide is more than the gain in the other, and a coupling constant that is periodically perturbed. This system manifests PIEP. Changing the coupling constant leads to an increase in total power limited only by nonlinear effects. Moreover, we show that the transmission coefficient of such waveguides is larger than in a system with constant parameters. Near the EP, we have a wide range of changes in parameters at which parametric instability occurs (Fig. 4). These results correspond to the feature of parametric instability mentioned above.

The phenomenon of PIEP may be used in metamaterial, plasmonic, and nanooptic devices whose applicability is substantially restricted by losses.

## Methods

In optical systems that consist of coupled waveguides or coupled microcavities, the power of the signal is defined as^6^,^35^ 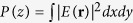. In the waveguide system with two eigenmodes *ϕ*_1_(*x*, *y*) and *ϕ*_2_(*x*, *y*), the power of the signal is given by the equation


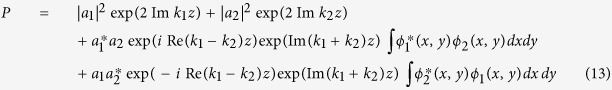


Here, we use the normalization 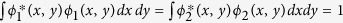. In the case of modes of waveguide systems, *P* is the power of the signal propagating along the waveguides, and *z* is the coordinate along the waveguide axis^6^,^17^,^48^. In the case of optical modes of a microcavity, *P* is power, and *z* is time^35^.

In a Hermitian system, 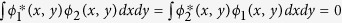 and Im*k*_1_ = Im*k*_2_ = 0, so the power of the signal is determined by the power of each mode and does not depend on the coordinate:


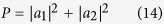


However, in a non-Hermitian system, 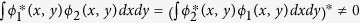, and the power of the signal is determined not only by the mode amplitudes but also by mode overlapping. By introducing *k* = Re(*k*_1_ − *k*_2_)^5^,^6^, we can rewrite Eq. (13) in the form


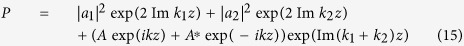


where 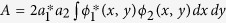.

## Additional Information

**How to cite this article**: Zyablovsky, A. A. *et al*. Parametric instability of optical non-Hermitian systems near the exceptional point. *Sci. Rep.* **6**, 29709; doi: 10.1038/srep29709 (2016).
